# Effect of empathy trait on attention to faces: an event-related potential (ERP) study

**DOI:** 10.1186/1880-6805-33-4

**Published:** 2014-01-24

**Authors:** Damee Choi, Shigeki Watanuki

**Affiliations:** 1 Department of Kansei Science, Kyushu University, 4-9-1, Shiobaru, Minami-ku, Fukuoka 815-8540, Japan; 2 Faculty of Design, Kyushu University, 4-9-1, Shiobaru, Minami-ku, Fukuoka 815-8540, Japan

**Keywords:** Empathy, Late positive potential, Event-related potential, Attention, Face

## Abstract

**Background:**

Empathy is deeply linked with the ability to adapt to human social environments. The present study investigated the relationship between the empathy trait and attention elicited by discriminating facial expressions.

**Methods:**

Event-related potentials were measured while 32 participants (17 men and 15 women) discriminated facial expressions (happy or angry) and colors of flowers (yellow or purple) under an oddball paradigm. The empathy trait of participants was measured using the Interpersonal Reactivity Index (Davis, 1980).

**Results:**

The empathy trait correlated positively with both the early portion (300 to 600 ms after stimulus onset) and late portion (600 to 800 ms after stimulus onset) of late positive potential (LPP) amplitude elicited by faces, but not with LPP elicited by flowers.

**Conclusions:**

This result suggests that, compared to people with low empathy, people with high empathy pay more attention when discriminating facial expressions. The present study suggests that differences exist in methods of adapting to social environments between people with high and low empathy.

## Background

Empathy is defined as ‘the ability to imagine oneself in another’s place and understand the other’s feelings, desires, ideas, and actions’ (Encyclopedia Britannica, 1999 edition). Empathy lets the individual understand another person’s emotions, such as pain, and to act altruistically toward that person [1]. Empathy also enables individuals to put themselves in the other person’s position, and to predict how they might act [2]. Some animals (for example, chimpanzees and dogs) also can take perspective of others; however, humans have more sophisticated and extensive empathy ability [3]. Empathy is thus an essential ability required for social activities of human being.

Individual differences exist in the empathy trait. In other words, sensitivity to sharing the emotions of others and the willingness to consider the positions of others vary among individuals. The reason why individual differences exist in empathy trait might be that the ability and methods to adapt to social environments differ depending on the individual. As mentioned above, the empathy ability of humans appears relatively sophisticated [3] and human social interactions are also complex. The complexity of human social interactions might thus cause individual differences in empathy trait.

Numerous studies have developed questionnaires to measure these individual differences in empathy trait. The Interpersonal Reactivity Index (IRI) [4] is one such questionnaire. As empathy has both a cognitive aspect (perspective-taking capabilities) and an emotional aspect (sensitivities to the emotions of another), the IRI was designed to measure these aspects separately [4]. The IRI comprises four empathy subscales: perspective taking; fantasy; empathic concern; and personal distress. The perspective taking scale measures attempts to take the perspectives of others, whereas the fantasy scale assesses the tendency to identify with fictitious characters [4]. The empathic concern scale measures the tendency to feel warmth and compassion for others, whereas the personal distress scale assesses the discomfort elicited by observing the negative experiences of others [4]. The IRI is widely used to measure the empathy trait not only in psychology, but also in neuroscience.

Neuroscience studies conducted over the past decade have shown that brain activity from stimuli containing human figures differs between people shown by the IRI to have high levels of empathy and those shown to have low levels [5-9]. For example, individuals with higher scores on the IRI show increased activation of the anterior insula and frontal operculum when observing the facial expressions of others [7]. These areas are said to be related to empathy and to be the part of the brain that becomes activated during empathy [9-11]. In addition, people with higher scores on the IRI exhibited stronger stress recovery effects in response to images of people showing pleasant emotions (for example, depicting familial love) after watching discomfort-inducing images [5]. These studies all indicate that people with high empathy are more sensitive to stimuli containing human figures. This raises the possibility that individuals with high empathy might pay more attention than those with low empathy when discriminating facial expressions. This is because discriminating facial expressions of others is important for understanding the emotional state and intention of others. However, whether the empathy trait correlates with attention elicited by discriminating facial expressions remains unclear, although the relationship between the empathy trait and brain activation elicited by just watching facial expressions was reported in a previous study [7].

The present study focused on late positive potential (LPP), an event-related potential (ERP) component that reflects the motivational significance of emotional stimuli [12-15]. The LPP is a positive slow wave beginning about 200 ms after stimulus onset and appearing maximal at centroparietal sites [12,15-18]. Many ERP studies have reported that LPP amplitude is greater in response to emotionally arousing (positive or negative) pictures than in response to emotionally neutral pictures [12,14,16,19,20]. Furthermore, LPP amplitude correlates positively with the subjective arousal level of pictures [12]. Thus, if individuals with high empathy pay attention to human faces more than those with low empathy, greater LPP is hypothesized to result when discriminating facial expressions. In addition, the later LPP (>600 ms) seems to represent a different component to the earlier LPP (<600 ms, defined as P3 or P300 in some studies) [16,21]. Recent studies have suggested that earlier LPP reflects obligatory capture of attention, whereas later LPP reflects elaborate processing and sustained attention [17,22,23]. This suggests the necessity of analyzing earlier LPP and later LPP separately.

‘Human’ elements such as human faces and voices provide important cues for triggering empathy. We can thus predict that, when presented with a stimulus not containing human figures, individuals with high empathy and those with low empathy should not attend to the stimulus differently. This hypothesis also appears plausible given the results of a study in which there was no difference in physiological stress recovery effects between those who scored high and those who scored low on the IRI when shown pleasant images not depicting human figures such as natural scenery [5]. We can thus predict that no difference would exist between individuals with high empathy and those with low empathy in the LPP response elicited by discriminating colors of flowers or other stimuli that do not depict a human presence.

We therefore examined the relationship between empathy trait (as measured by the IRI) and the LPP elicited by discriminating facial expressions, to identify whether individuals with high empathy pay attention in a different manner to those with low empathy when discriminating facial expressions. Participants discriminated images of faces (happy or angry) and flowers (yellow or purple) presented in oddball paradigm. We predicted that individuals with higher empathy would show greater amplitude of the LPP in response to faces, but not in response to flowers, reflecting enhanced attention only to stimuli containing human figures among individuals with higher empathy.

## Methods

### Participants

Thirty-two Japanese university or graduate school students participated in the study (17 men and 15 women; age range, 19–28 years; all right-handed). Participants completed the Japanese version [24] of the IRI, providing responses on a scale of 1 to 4 (does not describe me well: 1, to describes me very well: 4). After receiving an explanation of the purpose and details of the study, participants provided written informed consent prior to participation. The study protocols were approved by the ethics committee in the Department of Design at Kyushu University, Japan.

### Stimuli and procedure

For images of human faces, images of 12 adult humans (6 men and 6 women) showing two types of facial expression (happy or angry) were taken from the Karolinska Directed Emotional Faces [25], for a total of 24 images. Images of flowers were taken from the Internet. Twelve different images of flowers in two colors (yellow and purple) were selected (24 images in total). All images of human faces and flowers were edited to 300 × 400 pixels and presented in the center of a black screen (17-inch monitor, 1,024 × 768 resolution).

The experiment comprised four blocks of oddball tasks. In Block 1, the target was a happy face image, and the non-target was an angry face image. In Block 2, the target was a yellow flower image, and the non-target was a purple flower image. In Blocks 3 and 4, targets and non-targets of Blocks 1 and 2 were reversed. Participants were instructed to press a key with the right hand as soon as they saw the target. They therefore discriminated facial expressions (happy or angry) in Blocks 1 and 3, and flower colors (yellow or purple) in Blocks 2 and 4. Each block consisted of 60 trials, during which the target was presented 20% of the time (12 trials). In each block, each target image was shown once, and each non-target image was shown four times. Trials began with a 500-ms presentation of a cross shape followed by a random 800-ms presentation of a target or non-target image. Targets were never presented on two consecutive trials. Trials were separated by a 1,000-ms interval. This experimental design was based on that described by Fishman et al. [26].

After oddball tasks were completed, participants filled out subjective assessments. They once again observed the images presented in the oddball tasks, and judged the valence and arousal of each image based on a 7-point Likert scale (for valence, ‘very pleasant’ was given 3 points and ‘very unpleasant’ -3 points; for arousal, ‘very aroused’ was given 3 points and ‘very relaxed’ -3 points).

### ERP measurements and analysis

Electroencephalography (EEG) was recorded using a Polymate AP1532 system (TEAC co., Tokyo, Japan). Measurement sites were Cz (medial central), Pz (medial parietal), and Oz (medial occipital) sites based on the International 10–20 system [27], with averaged ears as reference. In the analysis, we focused on Cz and Pz sites because the LPP has shown to be maximal at the centroparietal site (for example, [12,15,16,23]). Electrooculography (EOG) was recorded to detect blinking with electrodes above and below the right eye. All electrode impedances were below 10 kΩ.

The EMSE Suite (Source Signal Imaging, San Diego, CA, USA) was used for ERP analysis. EEG signals were recorded at a sampling rate of 250 Hz and filtered with a low-frequency cutoff of 0.1 Hz and a high-frequency cutoff of 40 Hz. Blinking was corrected with the EMSE Ocular Artifact Correction Tool (for details, see [28]). Trials containing artifacts of 50 μV and trials during which the subject did not press a key were excluded from averages. Stimulus presentation of -200 to 800 ms was averaged (baseline: stimulus presentation of -200 to 0 ms) for four categories as follows: face targets, Blocks 1 and 3; flower targets, Blocks 2 and 4; face non-targets, Blocks 1 and 3; and flower non-targets, Blocks 2 and 4. The mean number of trials was 22.7 (standard deviation (SD) = 1.5) for face targets, 22.8 (SD = 2.3) for flower targets, 88.4 (SD = 9.0) for face non-targets, and 87.6 (SD = 14.3) for flower non-targets.

Early LPP was quantified as mean amplitude in the 300 to 600 ms after stimulus onset and late LPP was quantified as mean amplitude in the 600 to 800 ms after stimulus onset.

### Statistical analysis

SPSS version 17.0 software (SPSS, Chicago, IL, USA) was used for statistical analysis. Statistical significance was at a level of 5% (*P* < 0.05). We analyzed male and female data together, since no significant gender differences were apparent in IRI score (independent t-test, equal variances assumed; total IRI score, t = -0.92; Perspective taking, t = -0.69; Fantasy, t = -0.69; Empathic concern, t = -1.31; Personal distress, t = -0.20, all df = 30, *P* > 0.05).

Behavioral responses (response accuracies, reaction times, and subjective ratings) and LPP (early LPP and late LPP) were subjected to paired t-testing for comparisons between the two stimulus types (faces *vs.* flowers). To investigate relationships between empathy trait and responses to stimuli, we conducted Pearson correlation analysis between IRI score (total score and scores of the four subscales) and LPP.

## Results

### Empathy trait

Table 1 shows the IRI scores of participants.

**Table 1:** The range and mean (SD) of empathy trait (IRI score)

	**Range**	**Mean (SD)**
Total score	60-95	79.0 (10.2)
Perspective taking	14-26	20.4 (3.3)
Fantasy	12-27	20.9 (4.1)
Empathic concern	14-26	20.5 (2.9)
Personal distress	11-22	17.2 (2.8)

*n* = 32.

### Behavioral responses

Response accuracies were significantly (paired t-test, t = -3.96, df = 31, *P* < 0.001) higher in response to flowers (mean = 99.7%, SD = 0.7%) than in response to faces (mean = 98.6%, SD = 1.5%). Reaction times were significantly (paired t-test, t = 12.80, df = 31, *P* < 0.001) longer in response to faces (mean = 424.2 ms, SD = 70.7 ms) than in response to flowers (mean = 327.3 ms, SD = 38.5 ms). IRI results did not correlate significantly with response accuracies or reaction times (all *P* > 0.05, Pearson’s correlation coefficient).

For subjective ratings, valence rating indicated that flowers (mean = 0.9, SD = 0.9) were rated as significantly (paired t-test, t = -6.03, df = 31, *P* < 0.001) more pleasant than faces (mean = -0.2, SD = 0.4). Arousal rating showed that faces (mean = 1.3, SD = 0.9) were significantly (paired t-test, t = -7.05, df = 31, *P* < 0.001) more arousing stimuli compared with flowers (mean = -0.1, SD = 0.9). IRI did not correlate significantly with subjective ratings (all *P* > 0.05, Pearson’s correlation coefficient).

### LPP

**Figure 1:**
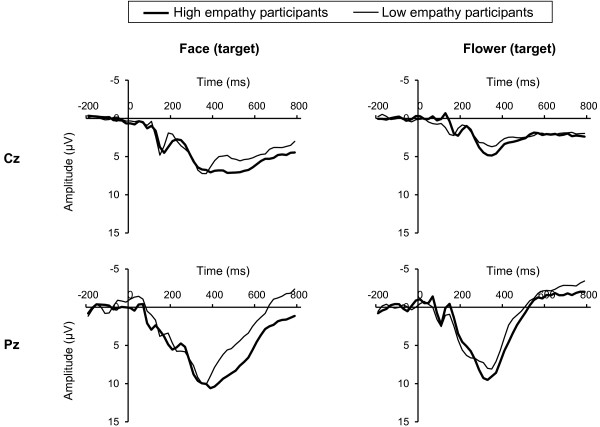
**Grand-averaged ERP waveforms elicited by targets.** High empathy participants (thick line, *n* = 18, range of total score of IRI: 82–95) and low empathy participants (thin line, *n* = 14, range of total score of IRI: 60–75) were selected by total IRI score and labelled only for illustrative purposes. Left and right column shows ERP waveforms elicited by face targets and flowers targets, respectively. Upper row shows Cz site and down row shows Pz site.

**Figure 2:**
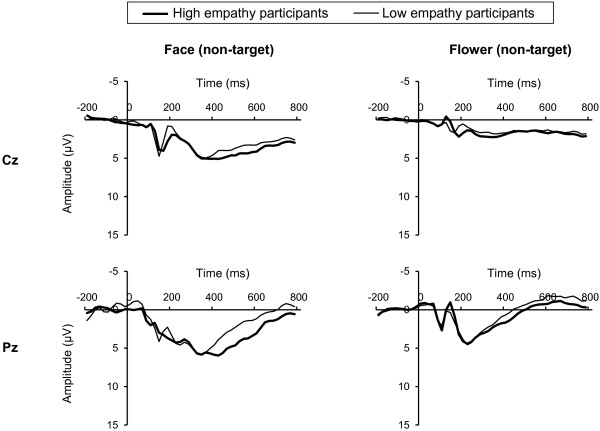
**Grand-averaged ERP waveforms elicited by non-targets.** High empathy participants (thick line, *n* = 18, range of total score of IRI: 82–95) and low empathy participants (thin line, *n* = 14, range of total score of IRI: 60–75) were selected by total IRI score and labelled only for illustrative purposes. Left and right column shows ERP waveforms elicited by face non-targets and flowers non-targets, respectively. Upper row shows Cz site and down row shows Pz site.

Figures 1 and 2 show grand-averaged ERP waveforms elicited by targets and non-targets, respectively.

**Figure 3:**
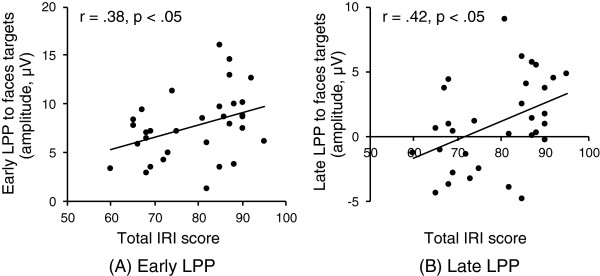
**Correlations between empathy trait (IRI score) and LPP elicited by faces targets (Pz site).** Total IRI score correlated positively with both **(A)** early LPP (300 to 600 ms) and **(B)** late LPP (600 to 800 ms) elicited by face targets at Pz site (all *P* < 0.05, Pearson’s correlation coefficient).

The early LPP was significantly greater in response to faces than in response to flowers (paired t-test, targets at Cz: t = 6.58, targets at Pz: t = 8.89, non-targets at Cz: t = 7.19, non-targets at Pz: t = 9.64, all df = 31, *P* < 0.001). Correlations between IRI scores and early LPP are shown in Table 2. Total IRI score showed a significant positive correlation with early LPP elicited by face targets at the Pz site (Pearson correlation, r = 0.38, *P* < 0.05) (Table 2, Figure 3A). For flower stimuli, early LPP did not show any significant correlation with IRI (all *P* > 0.05) (Table 2).

**Table 2:** Correlations between empathy trait (IRI score) and early LPP (300 to 600 ms)

**Early LPP**	**Face**				**Flower**			
**IRI score**	**Target**		**Non-target**		**Target**		**Non-target**	
	**Cz**	**Pz**	**Cz**	**Pz**	**Cz**	**Pz**	**Cz**	**Pz**
Total score	0.29	0.38^a^	0.16	0.26	0.11	0.19	-0.03	0.00
Perspective taking	0.09	0.27	0.12	0.23	-0.01	0.21	-0.18	-0.10
Fantasy	0.30	0.33	0.11	0.22	0.14	0.16	0.21	0.22
Empathic concern	0.28	0.27	0.20	0.17	0.05	0.06	-0.09	-0.16
Personal distress	0.20	0.31	0.06	0.18	0.15	0.15	-0.12	-0.03

*n* = 32.

^a^Pearson’s correlation coefficient; *P* < 0.05.

Late LPP was also significantly greater in response to faces than in response to flowers (paired t-test, targets at Cz: t = 4.74, targets at Pz: t = 6.73, non-targets at Cz: t = 3.47, non-targets at Pz: t = 6.15, all df = 31, *P* < 0.005). Correlations between IRI scores and late LPP are shown in Table 3. Total IRI score showed a significant positive correlation with late LPP elicited by face targets at the Pz site (Pearson correlation, r = 0.42, *P* < 0.05) (Table 3, Figure 3B). Furthermore, as shown in Table 3, the fantasy subscale of IRI correlated significantly and positively with late LPP elicited by face targets (Pearson correlation, Cz: r = 0.41, *P* < 0.05, Pz: r = 0.47, *P* < 0.01) and face non-targets at the Pz site (Pearson correlation, r = 0.36, *P* < 0.05). In addition, the personal distress subscale of IRI also showed a significant positive correlation with late LPP elicited by face targets at the Pz site (Pearson correlation, r = 0.38, *P* < 0.05) (Table 3). For flower stimuli, late LPP did not show any significant correlation with IRI (all *P* > 0.05) (Table 3).

**Table 3:** Correlations between empathy trait (IRI score) and late LPP (600 to 800 ms)

**Late LPP**	**Face**				**Flower**			
**IRI score**	**Target**		**Non-target**		**Target**		**Non-target**	
	**Cz**	**Pz**	**Cz**	**Pz**	**Cz**	**Pz**	**Cz**	**Pz**
Total score	0.30	0.42^a^	0.13	0.17	0.07	0.13	-0.05	0.02
Perspective taking	0.00	0.21	0.07	0.08	-0.20	-0.06	-0.14	-0.04
Fantasy	0.41^a^	0.47^b^	0.21	0.36^a^	0.24	0.24	0.22	0.28
Empathic concern	0.31	0.19	0.13	-0.03	0.02	-0.01	-0.12	-0.19
Personal distress	0.17	0.38^a^	-0.06	0.04	0.12	0.19	-0.22	-0.11

*n* = 32.

^a^Pearson’s correlation coefficient; *P* < 0.05.

^b^*P* < 0.01.

Correlations between IRI score and LPP elicited by faces were compared with correlations between IRI score and LPP elicited by the flowers, using Fisher’s test. However, no significant differences were identified (all z <1.64, *P* > 0.05).

## Discussion

The goal of the present study was to investigate whether individuals with high empathy pay attention when discriminating facial expressions differently from those with low empathy. We examined the relationship between empathy trait (IRI) and LPP.

Consistent with our hypothesis, participants with higher IRI score showed larger LPP in response to faces. This result was shown not only when faces were presented as targets, but also when faces were presented as non-targets. This indicates that individuals with high empathy pay attention to human faces more than individuals with low empathy when discriminating facial expressions. We argue that this is because individuals with high empathy tend to try to correctly gauge the emotional state and intentions of other people more than individuals with low empathy. There thus seem to be distinct differences in the methods of adapting to social environments between individuals with high and low empathy. In addition, we analyzed early LPP (300 to 600 ms) and late LPP (600 to 800 ms) separately and found that both correlated positively with total IRI score. This indicates that empathy is associated not only with obligatory capture of attention by faces, but also with elaborate and sustained processing of faces. The present findings thus suggest empathy trait as one of the factors eliciting individual differences in the processing of faces.

The relationship between empathy trait and LPP can also be explained from the interaction of bottom-up and top-down processing in empathic responses. Empathy is influenced by both automatic unconscious bottom-up processes and top-down processes that involve voluntary control (reviewed in [29,30]). For example, when we watch a movie and see the face of an actor who has been frightened by something, we sense fear unconsciously through bottom-up processes, but can simultaneously consciously control that fear through top-down processes. As stated in the Introduction, many neuroscience studies have reported that the empathy trait affects brain activity elicited by stimuli containing human figures [5-9]. However, whether differences in brain activity due to the empathy trait are caused by bottom-up processing or top-down processing of empathy remains unclear (reviewed in [30]).

LPP appears to reflect the interaction of bottom-up and top-down processing in response to stimuli [18,31,32]. As stated in the Introduction, LPP is greater in response to emotionally arousing pictures than in response to emotionally neutral pictures [12,14,16,19,20]. This suggests that LPP is associated with bottom-up processing, such as automatic responses to emotional stimuli. On the other hand, LPP has also been argued to index top-down processing such as voluntary modulation of emotion or attention. For instance, a smaller LPP is elicited when suppressing emotional responses to arousing pictures compared with when watching the pictures normally and when enhancing emotional responses to the pictures [33]. In addition, mean amplitude in the 360 to 800 ms range (defined as P3 in the study) is greater when rating the pain in an image (for example, an image of someone getting their finger caught in a door) compared with when simply counting the number of fingers in an image [34]. Thus, the present results showing the relationship between empathy trait and LPP elicited by faces could be interpreted as showing that both bottom-up and top-down processing of empathy affect individual differences in responses to stimuli containing human figures.

In addition, we found that late LPP elicited by faces correlated positively with fantasy and personal distress scales, but not with perspective taking or empathic concern scales. This is consistent with the findings of Jabbi et al. [7], who demonstrated that the fantasy and personal distress scales showed stronger correlations with activation of the anterior insula and frontal operculum elicited by observing facial expressions than other subscales of the IRI. Personal distress has been suggested to be associated with self-oriented empathic response (that is, imagining oneself to be in the situation of others), while empathic concern is associated with other-oriented empathic response (that is, imagining the feelings of others) [35]. In addition, the fantasy scale seems to reflect self-oriented empathic response more than other-oriented empathic response, given that it measures the tendency to identify with characters in fictional situations [4]. The present study suggests that responses to faces are associated with self-oriented empathic response, as reflected in the personal distress and fantasy scales of the IRI.

In the present study, images of flowers were presented as stimuli that do not contain human figures. As hypothesized, in response to images of flowers, IRI scores did not correlate with LPP. This indicates that no difference exists in attention when discriminating colors of flowers between individuals with high and low empathy. This may mean that, compared to individuals with low empathy, those with high empathy have a higher tendency to pay particular attention to human elements among the various stimuli they encounter. However, this interpretation has some limitations. First, the LPP was smaller in response to flowers compared with faces, reflecting that degree of attention to flowers was not the same as that to faces. Second, subjective ratings also revealed that images of faces were more arousing stimuli than images of flowers. The low motivational value of flowers might thus have caused similar ERP responses between individuals with high and low empathy. Third, correlations between IRI scores and LPP elicited by faces were not significantly stronger than those correlations between IRI scores and LPP elicited by flowers. Finally, results of response accuracies and reaction times indicate that discriminating facial expressions was more difficult than discriminating colors of flowers. Thus, in further research, it is necessary to compare LPP elicited by faces and LPP elicited by stimuli with equal difficulty of discrimination without human figure.

## Conclusions

The present study revealed that empathy trait (as determined using the IRI) correlated positively with both early (300 to 600 ms) and late (600 to 800 ms) portions of LPP elicited when discriminating facial expressions, but not colors of flowers. This indicates that individuals with high empathy pay more attention when discriminating facial expressions than individuals with low empathy. The present findings suggest that differences exist in the methods of adapting to social environments between individuals with high and low empathy.

## Abbreviations

ERP: Event-related potential; IRI: Interpersonal reactivity index; LPP: Late positive potential.

## Competing interests

The authors declare that they have no competing interests.

## Authors’ contributions

DC and SW contributed to the design of the experiment. DC performed the experiments, analyzed the data, and wrote the manuscript with advice of SW. Both authors read and approved the final manuscript.
